# Urban growth simulation in different scenarios using the SLEUTH model: A case study of Hefei, East China

**DOI:** 10.1371/journal.pone.0224998

**Published:** 2019-11-07

**Authors:** Yunqiang Liu, Long Li, Longqian Chen, Liang Cheng, Xisheng Zhou, Yifan Cui, Han Li, Weiqiang Liu

**Affiliations:** 1 School of Environmental Science and Spatial Informatics, China University of Mining and Technology, Xuzhou, Jiangsu, China; 2 Engineering Research Center of Ministry of Education for Mine Ecological Restoration, China University of Mining and Technology, Xuzhou, Jiangsu, China; 3 Department of Geography, Earth System Science, Vrije Universiteit Brussel, Brussels, Belgium; 4 College of Yingdong Agricultural Science and Engineering, Shaoguan University, Shaoguan, Guangdong, China; Institute of Geographic Sciences and Natural Resources Research (IGSNRR), Chinese Academy of Sciences (CAS), CHINA

## Abstract

As uncontrolled urban growth has increasingly challenged the sustainable use of urban land, it is critically important to model urban growth from different perspectives. Using the SLEUTH (Slope, Land use, Exclusion, Urban, Transportation, and Hill-shade) model, the historical data of Hefei in 2000, 2005, 2010, and 2015 were collected and input to simulate urban growth from 2015 to 2040. Three different urban growth scenarios were considered, namely a historical growth scenario, an urban planning growth scenario, and a land suitability growth scenario. Prediction results show that by 2040 urban built-up land would increase to 1434 km^2^ in the historical growth scenario, to 1190 km^2^ in the urban planning growth scenario, and to 1217 km^2^ in the land suitability growth scenario. We conclude that (1) exclusion layers without effective limits might result in unreasonable prediction of future built-up land; (2) based on the general land use map, the urban growth prediction took the governmental policies into account and could reveal the development hotspots in urban planning; and (3) the land suitability scenario prediction was the result of the trade-off between ecological land and built-up land as it used the MCR -based (minimum cumulative resistance model) land suitability assessment result. It would help to form a compact urban space and avoid excessive protection of farmland and ecological land. Findings derived from this study may provide urban planners with interesting insights on formulating urban planning strategies.

## Introduction

Recent decades have seen an accelerating urban growth on a global scale. By 2050, the average urbanization rate is expected to reach 86% in developed countries, and 64% in developing countries [1]. Since 1978, the beginning of its reform and opening-up policy, China has been urbanized at an unprecedented pace. China’s urbanization rate was only 20.1% in 1981 while this figure rose to 57.4% in 2016. Such high-speed urban growth is considered responsible for a variety of issues such as climate change, ecosystem services degradation, and farmland loss, and challenging to the sustainable use of urban land [2,3]. It is therefore of great practical significance to coordinate the contradiction between urbanization and ecological protection and establish a model to simulate and analyze urban growth.

There exist a variety of urban growth simulation models, such as logistical regression [4–6], and agent-based model [7], cellular automata (CA) [8]. The logistic regression model can well explain the relationship between urban growth and various driving factors [4]. However, it is not explicit in time and does not account for the correlation between driving factors [9]. Although the agent-based modeling has satisfactory applicability in urban growth simulation, the uncertainty of the initial conditions and the behavioral rules of the agent can produce highly variable simulation results [9,10]. Among all the models, CA is the most widely used urban growth modeling approach due mainly to its flexibility, visualization, and capability to integrate spatial and temporal processes and simulate complex dynamic systems [8,11]. In addition, CA models can directly use raster data and this allows easy integration with remote sensing and GIS technologies [12–16].

Several different CA-based urban models have been proposed for urban growth simulation, land use change simulation, and planning policy evaluation [17,18], such as UrbanSim [19], ANN -CA (artificial neural network) [20], SVM-CA (support vector machine) [21], RF-CA (random forest) [22], and SLEUTH model. The UrbanSim model has not been widely used as it integrates seven submodules and requires huge data support and high requirements for data organization [19]. Although the conversion rules of CA established by intelligent algorithms can explore the relationship between urban land use change and driving factors [23], the resulting conversion rules are only applicable to the local urban growth mode. Consequently, these models are not suited to define general urban CA [8]. The SLEUTH model, developed by Clarke et al. [24] with predefined conversion rules applied spatially to gridded maps of the cities in a set of nested loops [25], has been widely used to simulate the urban growth worldwide due to its universal applicability and refined over the past decades. In 2007, Dietzel and Clarke used self-organizing map theory to calibrate the model and proposed goodness of fit metric, OSM (Optimal SLEUTH Metric), which would provide the most robust results for SLEUTH calibration [26]. A new version of the SLEUTH model was developed in 2010 by introducing new fit metrics, expanding the capability of SLEUTH to incorporate policy information and increasing the speed of model calculations [27]. The SLEUTH model can help create different future urban growth scenarios by changing the input data or modifying the coefficients of the model [26,28,29]. In addition, the model can be used as an important planning tool to incorporate different urban development factors into future urban growth projections [30]. A number of studies have incorporated policy planning, environmental quality, and hydrological models into the model to achieve coupling of the SLEUTH model with other data sets or methods [31–33]. Given its success in urban growth simulation and capability of defining scenarios and integrating other data and models, we used the SLEUTH model in conjunction with policies and land assessment for the future urban growth studies.

Suitability evaluation of urban built-up land is critical for both urban growth and the proper utilization of land resources [34]. This study uses the minimum cumulative resistance (MCR) model to construct an urban land suitability evaluation system. The minimum cumulative resistance model regards the urban land use change as a horizontal movement of the existing construction and ecological land on the resistance surface, which is reflected in the competitive relationship between the built-up land and the ecological land during the urban growth process. It would be interesting if the result of the MCR-based ecological suitability evaluation could be integrated into the SLEUTH model as the exclusion layer.

The study selected the main urban area of Hefei as the study area. The input data of the SLEUTH model include urban built-up land layers and traffic road layers in 2000, 2005, 2010, and 2015, a slope layer, a hill-shade layer, and exclusion layers. By changing the exclusion layer of the SLEUTH model, three urban growth scenarios were developed, namely a historical growth scenario, an urban planning growth scenario, and a land suitability growth scenario. The historical growth scenario only excluded the water. The urban planning growth scenario incorporated the 2006–2020 general land use planning map. The land suitability growth scenario achieved loose coupling between the MCR model and the SLEUTH model. After the three scenarios were independently calibrated, and their optimal parameter combinations were identified, we predicted the urban growth of Hefei from 2015 to 2040 for each scenario. The ultimate objective of this study is to provide decision-makers and urban planners with interesting insights on formulating urban management and urban planning strategies through projections of urban growth in different scenarios.

## Study area and data

### Study area

**Fig 1:**
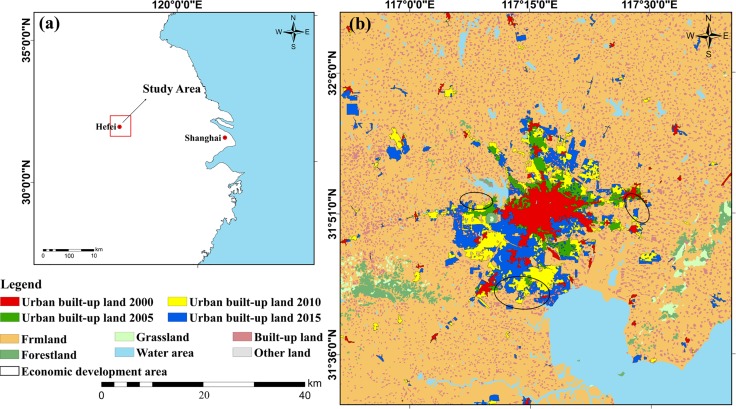
Study area. (a) Location of the study area in Anhui, China; (b) urban built-up land growth in Hefei in 2000, 2005, 2010, 2015.

Located in the center of east China’s Anhui province (30°57′N~32°32′N, 116°41′E~117°58′E) (Fig 1A), Hefei covers an area of 11408.48 km^2^ with an average altitude of 30 m. As the provincial capital, Hefei is also the cultural, commercial, financial, and political center of Anhui. It is characterized by a subtropical monsoon climate with an average annual temperature of 16°C and total annual precipitation of 995.2 mm. The prefectural city of Hefei now comprises four counties (Changfeng, Feidong, Feixi, and Lujiang), four districts (Yaohai, Luyang, Shusha, and Baohe), and one county-level city (Chaohu, once an Anhui’s prefectural city incorporated by Hefei in 2011).

Since 2000, a number of urban development strategies have been implemented, such as the major development, large construction, large development strategy in 2006, the “141” urban development strategy in 2007, and the “1331” development strategy in 2013, making Hefei’s economy and urban extent fast growing. Statistics from the 2000 official census show that the population and GDP (Gross Domestic Product) of Hefei in 2000 were 4.47 million and 369.16 billion RMB. These figures rose to 7.87 million and 5660.27 billion RMB in 2015, respectively. Over the 15 years, the built-up area grew by 250.56% from 125 km^2^ to 438 km^2^. Demand for built-up land continues to grow due to priority given to economic development and urban construction. Land resources and environmental capacity outnumber their supply [35]. Since limited land resources are hindering the sustainable development of urbanization, among Hefei's urgent tasks is to assess the suitability of construction sites and to make reasonable use of limited land resources. As the capital city of Anhui and one of the central cities in the Yangtze Delta Region, Hefei leads the urbanization process in this east Chinese province. In the context of the National New-type urbanization Plan [36], the experience of its urban development will provide valuable insights to its neighboring and similar cities. Therefore, we chose the urban growth process of Hefei from 2000 to 2015 as the case study.

### Data

Data used in this study includes multi-temporal Landsat datasets, Digital Elevation Model (DEM) data, traffic network vector data, the 2006–2020 land use planning map of Anhui province, soil type map and precipitation map. The Landsat datasets and DEM, both at a spatial resolution of 30 m, were obtained from the United States Geological Survey website (USGS, https://earthexplorer.usgs.gov/). The soil and precipitation maps were provided by the Data Center for Resources and Environmental Sciences, Chinese Academy of Sciences (RESDC, http://www.resdc.cn/). The four Landsat TM image data from 2000, 2005, 2010, and 2015 were used for landscape classification. After atmospherically corrected using the FLAASH (Fast Line-of-sight Atmospheric Analysis of Spectral Hypercubes) utility in ENVI 5.3 and geometrically corrected with total RMS (root mean squared) of less than half a pixel, they were classified into six broad categories using the support vector machine (SVM) supervised classifier, namely urban built-up land, farmland, forestland, grassland, water, and other land. The SVM is a group of theoretically superior machine learning algorithms [37], and has proven accurate for mapping urban land cover from medium-resolution imagery [38–40]. The SVM classifications in this study were implemented using an ENVI add-in known as EnMAP-box [41]. To assess classification accuracy for the 2010 and 2015 classification results, we used high-resolution Google Earth images, where a total of 400 sample points were randomly selected as reference data using ArcGIS 10.2’s Create Random Points tool. By the confusion matrices, the Kappa coefficients were both above 0.8 for the 2010 and 2015 classification results. Due to the lack of high-resolution remote sensing images, we were not able to perform independent classification accuracy assessments for the results of 2000 and 2005. However, as the images were all Landsat data and processed and classified using the same procedure and approach, we thus consider that similar classification accuracies were obtained for the 2000 and 2005 images [42]. As such, the Kappa coefficients (>0.8) we obtained indicates that the classification results were acceptable [43]. All raster and vector data were clipped to the same extent of the study area (Fig 1B), and the projection coordinate system was uniformly set to WGS_1984_UTM_Zone_50N.

## Methods

### SLEUTH model

The SLEUTH model consists of two sub-models: the urban growth model (UGM), which can be run independently, and land cover deltatron model (LCD) [15]. The SLEUTH model requires a minimum of four periods of city-wide layers, two periods of transportation layers, slope layers, hill- shade layers, and exclusion layers [29]. Based on grid cells, the SLEUTH model assigns the attributes of the city or non-city to each cell and simulates urban growth by four conversion rules—spontaneous growth, new spreading center growth, edge growth, and road-influenced growth [24]. Spontaneous growth defines the occurrence of random urbanization of land, i.e., randomly selected non-urbanized cells may be transformed into urbanized cells when slope conditions are appropriate. An urban spreading center is defined as a location with three or more adjacent urbanized cells. New spreading center growth determines whether any of the new, spontaneously urbanized cells would become new urban spreading centers. Edge growth defines the growth of an existing spreading center, which simulates the urban's fill-in growth and the expansion of boundaries [24,28]. Road-influenced growth encourages urbanized cells to develop along road network, which simulates the impact of existing transportation infrastructure on urban growth. The mentioned four urban growth rules are performed sequentially in each growth cycle and are controlled by five growth coefficients [44], namely dispersion coefficient, breed coefficient, spread coefficient, road gravity coefficient, and slope coefficient. The relationship between the four growth rules and the five coefficients is shown in Table 1.

**Table 1:** Relationship between growth rules and model coefficient in the SLEUTH model.

Growth rules	Model coefficients	Rule description
Spontaneous growth	Dispersion, slope	Random conversion of non-urban cell to urban cell
New spreading center growth	Breed, slope	Urban cell from spontaneous growth become new spreading centers
Edge growth	Spread, slope	Spreading center edge expansion
Road influenced growth	Breed, road-gravity, dispersion, slope	The attraction of traffic roads to urbanization

**Fig 2:**
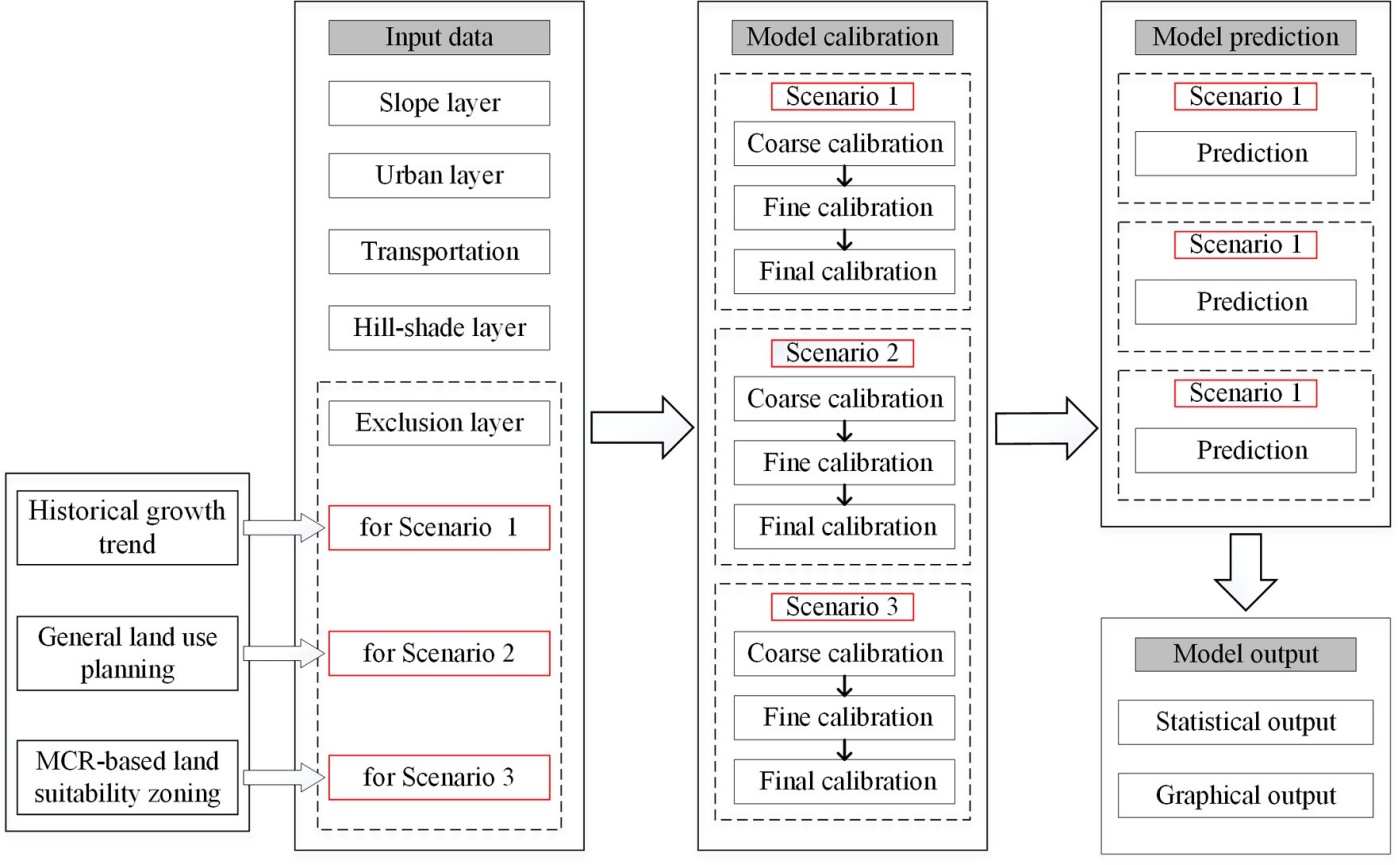
Technical flowchart of the study.

The execution of the SLEUTH model is divided into four major steps (Fig 2): input data preparation, model calibration, model prediction, and model output [45]. Three urban growth scenarios were planned by changing the exclusion layer, and both the calibration and prediction of the three scenarios were performed independently. We used the SLEUTH 3.0 beta_p01 module (http://www.ncgia.ucsb.edu/projects/gig/) and completed the model compilation and operation with the help of the software of Cygwin.

### Input data preparation

**Fig 3:**
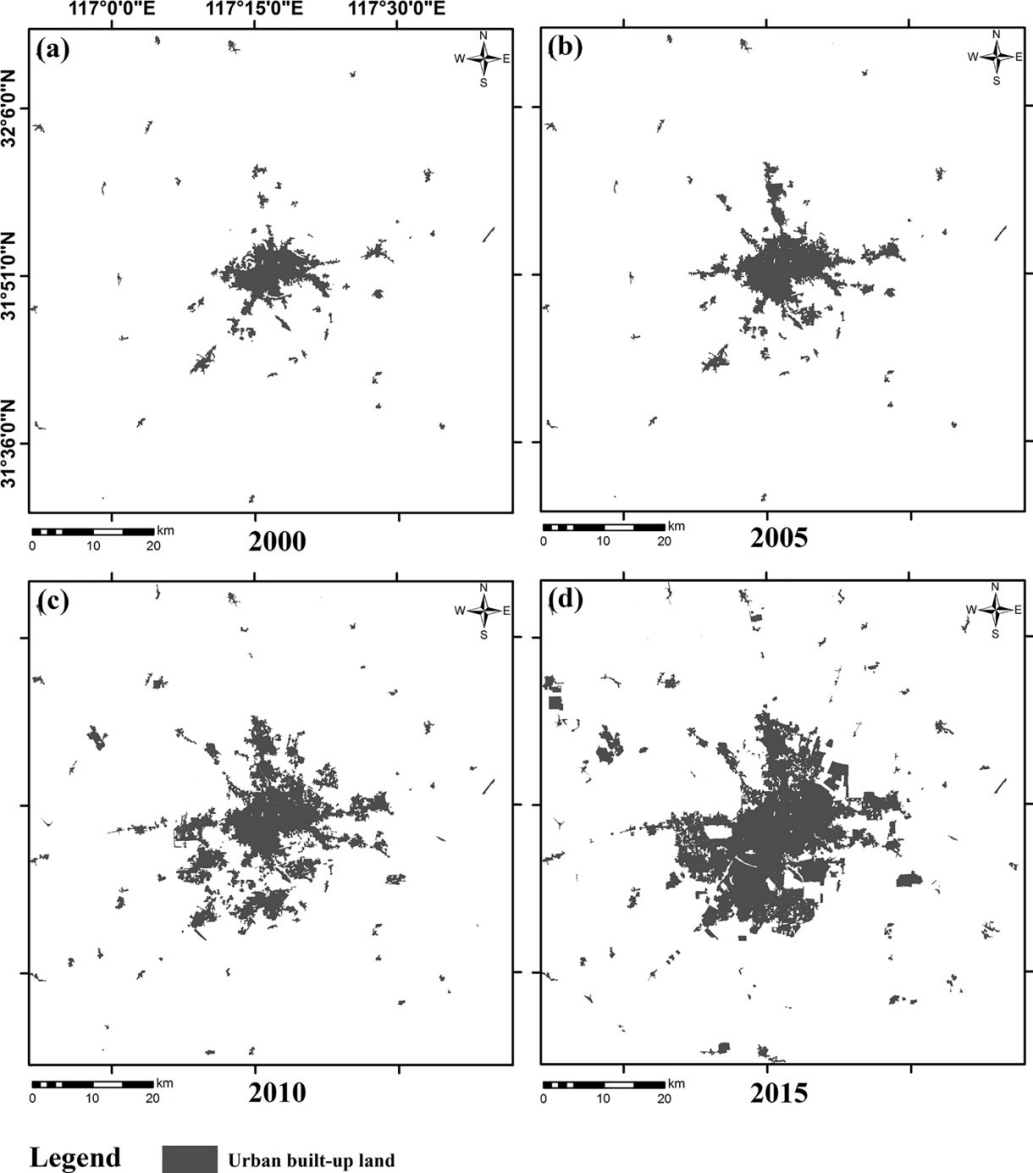
Inputs data: Urban built-up land of Hefei in 2000, 2005, 2010, and 2015.

**Fig 4:**
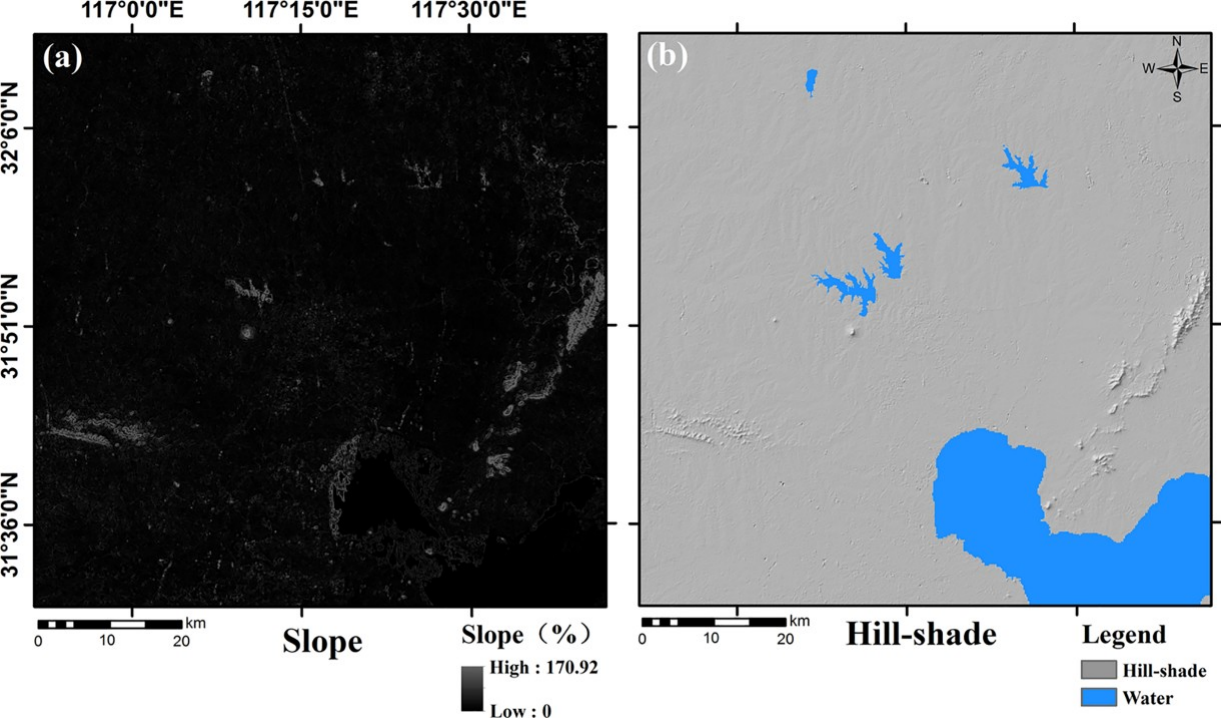
Input data: Slope layer and hill-shade layer.

**Fig 5:**
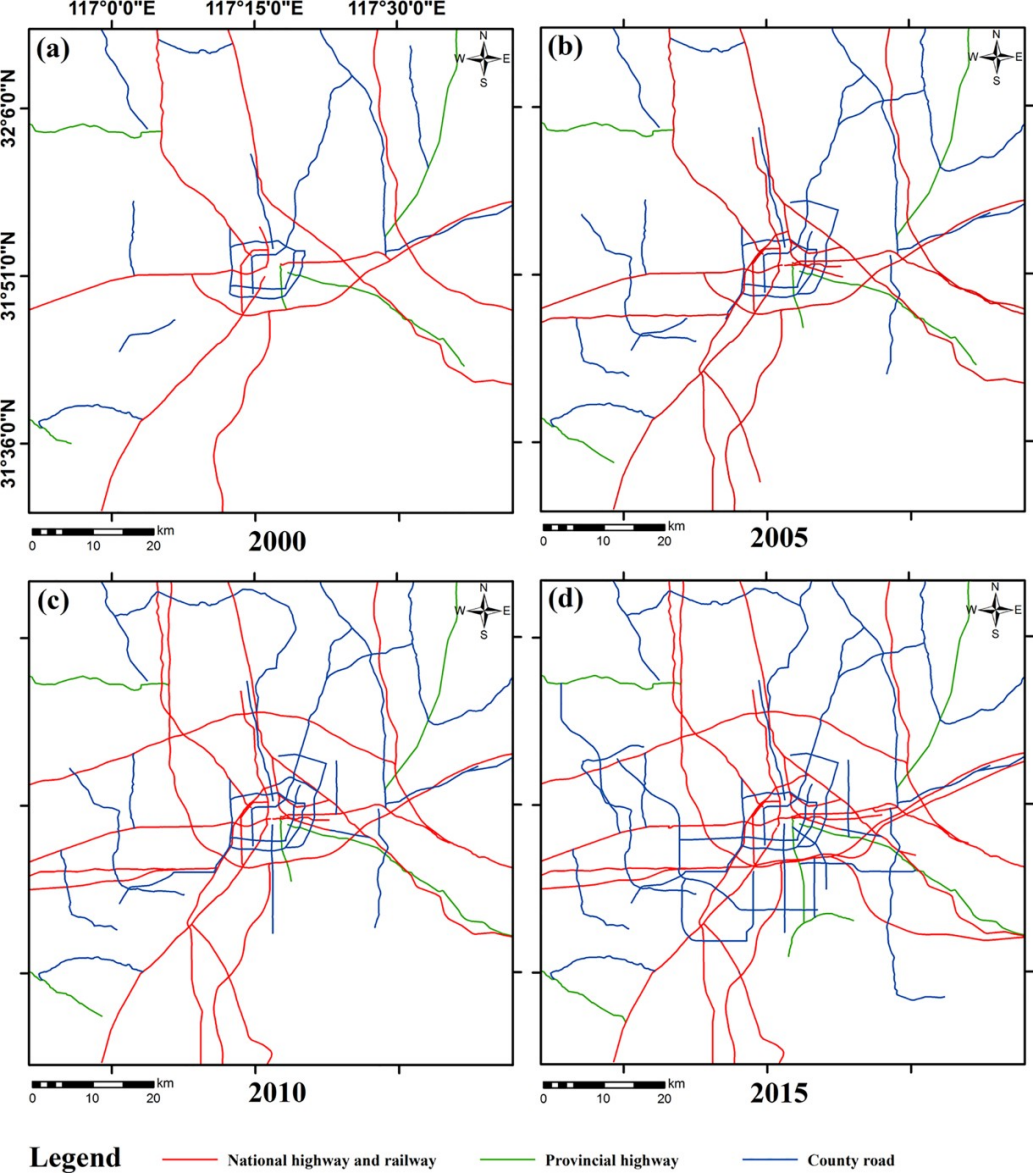
Input data: Road network of Hefei in 2000, 2005, 2010, and 2015.

The input data required by the SLEUTH model include the urban layer, the slope layer, the hill-shade layer, the transportation layer, and the exclusion layer. Since the study only activated the UGM module, the land use layer was not included in the input data. Through satellite image classification, we extracted the urban built-up land and used it as the urban layers for the years of 2000, 2005, 2010, and 2015 (Fig 3). Both the slope layer and the hill-shade layer were derived from the DEM, and the unit of the slope was percentage (Fig 4). The road network data was vectorized from the satellite images to produce the transportation layers, and the road grade information was determined using the traffic thematic map (Fig 5). As roads may have a different impact on urban growth [46], we divided roads into three levels and assigned different weights to them. A weight of 100 was given to national highways and railways as they are highly conducive to urban growth while the weight of provincial highways was set at 50 due to their medium influence. In addition, the weight of county roads and the non-road area were determined as 25 and 0 [29]. The exclusion layers were used to discriminate different urban growth scenarios. The spatial resolution of all input data was uniformly re-sampled to 60 m in order to reduce the computational workload. This research used the same spatial resolution in both the model calibration and prediction phases. As required by the SLEUTH model, all input layers were converted into grayscale GIF (graphics interchange format) images.

### Scenario setting

The range of gray values for the exclusion layer grid cells was set to 0–100, and the gray pixel value indicates the probability of the grid cells not being urbanized. Using the exclusion layer, users can define the probability of urbanization for different areas [26]. For example, the exclusion probability of the ocean would be defined as 100%, while forest parks would be defined as 75%. In this way, users could set up different urban growth scenarios.

The most straightforward practice is setting the exclusion probability for all areas at 0, though sometimes except for major water bodies [13,29,47]. Another approach is assigning different exclusion probabilities to land use type according to urban planning [13,25,48]. Among them, high exclusion probability can be set for nature reserves, forest areas and waters to reflect the protection of ecological land. These two methods were used for constructing two scenarios in this study for urban growth prediction. However, the two scenarios do not emphasize the competition between built-up land and ecological land, which plays a key role in urban growth. We, therefore, made use of the minimum cumulative resistance (MCR) model to generate a different exclusion layer for constructing the third scenario. This study set up three different urban growth scenarios: a historical growth scenario, an urban planning growth scenario, and a land suitability growth scenario.

**Fig 6:**
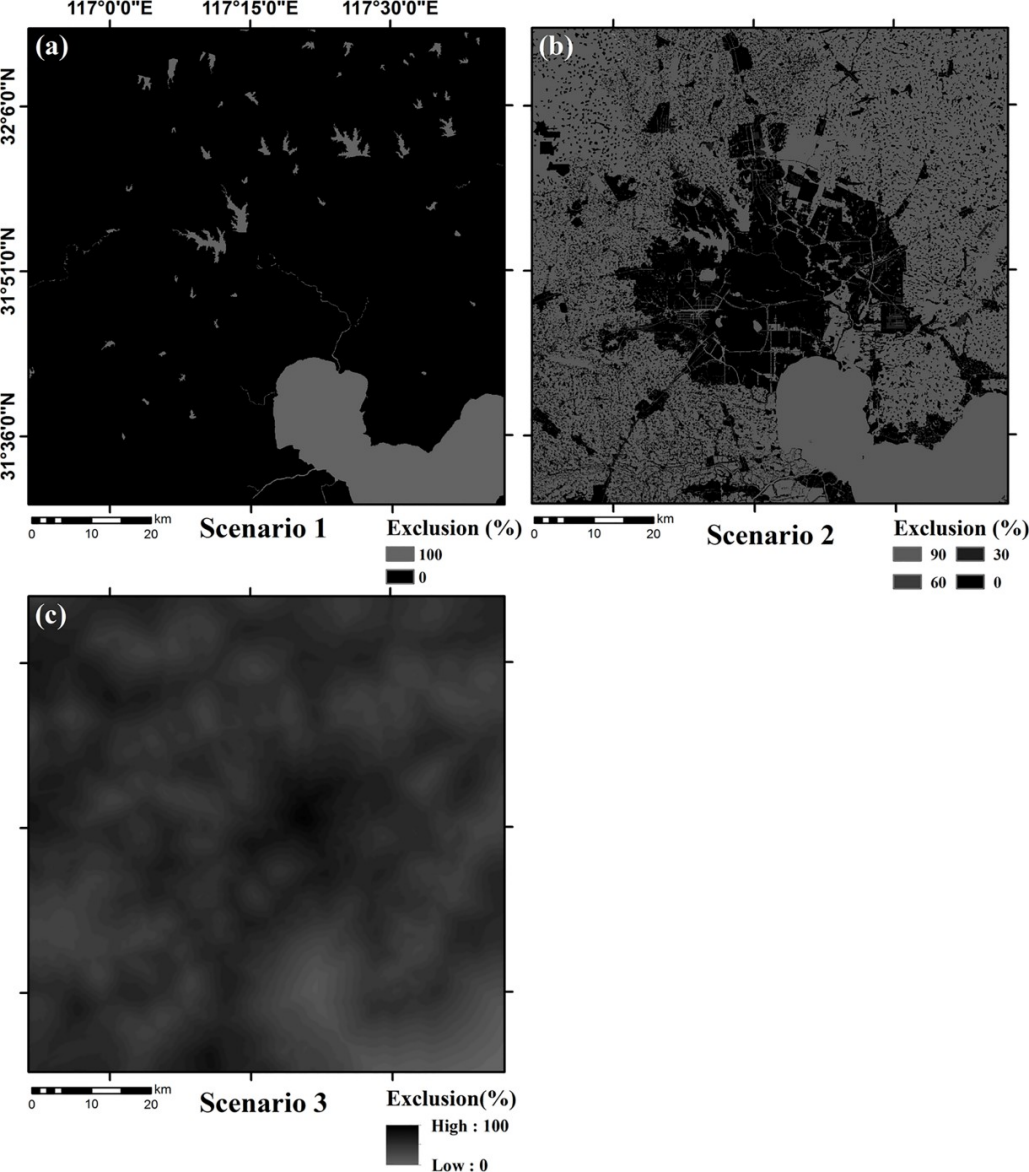
Input data: Exclusion layer for three scenarios. (a) Scenario 1, the historical growth scenario; (b) Scenario 2, urban planning growth scenario; (c) Scenario 3, land suitability growth scenario.

- Scenario 1: In the historical growth scenario, existing urban areas are expected to expand simply based on the previous growth pattern. Only large lakes and reservoirs were excluded (100% non-urbanization probability) and urbanization in other areas was not restricted (Fig 6A).
- Scenario 2: In the urban planning growth scenario on the 2006–2020 general land use planning map of Hefei is used as the basic data. By summarizing the influence of different land planning types on urban growth [39,41], each types of exclusion probability were determined. The exclusion probability of land permitted for construction (LPC; the land that is allowed to be used as construction land for urban and rural construction [49]) in urban areas and rural areas was defined as 0 while the exclusion probability of land conditionally permitted for construction (LCPC; the land where urban and rural construction can be carried out after meeting certain conditions [49]) was defined as 30%. The exclusion probability was defined as 60% for cultural heritage reserve, and 90% for basic farmland, forestry areas, animal husbandry areas, water areas, and natural reserves (Fig 6B).
- Scenario 3: In the land suitability growth scenario, the result of the MCR-based urban land suitability zoning was integrated into the exclusion layer. While detailed land suitability zoning would more objectively reflect the impact of land suitability on urban expansion and avoid overestimation or underestimation of suitability in certain areas, the computational cost would be considerably increased. As a comprise between prediction accuracy and computational cost, we, therefore, divided the urban land suitability zoning map into 20 grades according to the order of suitability from small to large. The area of each level was evenly assigned the corresponding gray value ranging from 0 to 100 (Fig 6C). The following sections provide a detailed description on producing the exclusion layer for the land suitability growth scenario.

#### Minimum cumulative resistance model

Originating from the study of dispersion processes of species [50], the minimum cumulative resistance refers to the minimum cost involved in the process of crossing a heterogeneous landscape between a source and a target and reflects the spatial accessibility between the source and the target [51,52]. Some researchers have recently applied the MCR model to the studies of urban spatial growth and land ecological suitability evaluation [21]. Traditional ecological suitability evaluation methods, such as map superposition and logical rule combination, superimpose the evaluation factors of landscape units and emphasize the vertical process of landscape units while ignoring the trend of various landscape horizontal directions [53]. The MCR model regards land expansion as horizontal spatial movements on the resistance surface. As such, it offset the disadvantage of the traditional methods, i.e., only considering vertical landscape evaluation. Proposed by Knaapen et al., this model was improved by Yu et al. [54], which is given by:

$$MCR=f_{min}\sum_{j=n}^{i=m}D_{ij}\times R_{i}$$

where MCR is the minimum cumulative resistance value, *f* is some unknown but monotonically decreasing function, *D*_*ij*_ and *R*_*i*_ represent the spatial distance and relative resistance value respectively when a species travels from source *j* through landscape type *i* to any point on a landscape surface [34,55]. The minimum cumulative resistance is obtained through the Cost Distance function in the ArcGIS’s Spatial Analyst tool.

#### Expansion source and resistance surface

In this study, we assumed that land could be divided into two broad categories: ecological land suitable for establishing natural ecosystems and built-up land suitable for urban facilities construction. The expansion of the two land types could be seen as a spatial movement on the ecological/built-up land expansion resistance surface.

a. Source selection. The source region with internal homogeneity and spatial expansion capability is the starting point for an expansion movement [56]. The source of ecological land expansion is set as land with rich biodiversity and ecosystem services, e.g., lakes, rivers, green spaces, and wetlands. The ecological land sources in the study area include Chaohu Lake, Wabu Lake, Dongpu reservoir, Dafang reservoir, Lushan Forest Park, Zipeng mountain forest park, and Fucha Mountain. The urban built-up land in 2015 was set as the source of built-up land expansion.

b. Resistance surface. A resistance value indicates the difficulty of converting a land type into another type under the influence of certain environmental factors [57]. The resistance value in the same environment varies with the type of expansion. The resistance value is a relative value rather than an absolute value. Five evaluation factors of topography, ecological function, landscape type, soil erosion sensitivity, and ecological value were selected to establish a landscape process resistance evaluation system [57–59]. The resistance value of each evaluation factor was divided into five levels, which were assigned 1, 2, 3, 4, and 5, respectively. A higher level means greater resistance to a certain expansion type. The expansion motion of the two sources was performed in the resistance plane of the same evaluation system, but the resistance values of the two resistance surfaces were opposite. As water and vegetation are essential indicators of ecological values, the normalized vegetation index (NDVI) and the distance from water bodies were analyzed using a classification matrix [57]. Soil erosion sensitivity accounted for slope, vegetation coverage, soil type, and precipitation. Areas with strong ecological functions such as nature reserve, forest parks, and basic farmland were given the high resistance to built-up land expansion. The resistance scores of each evaluation factor are shown in Table 2. In this study, the extremum method was used to form the ecological/built-up resistance surface. The minimum values of the five resistance factors were used as the expansion resistance of ecological land, and the maximum values were used as the expansion resistance of built-up land.

**Table 2:** The evaluation system for resistance factors to ecological land and built-up land. [53].

Ecological value		Resistance value of ecological land (Resistance value of built-up land)	Distance from adjacent water				
			0~50	50~100	100~150	150~200	>200
	NDVI	0.8~1.0	1 (5)	1 (5)	2 (4)	2 (4)	2 (4)
		0.6~0.8	1 (5)	2 (4)	2 (4)	3 (3)	3 (3)
		0.4~0.6	2 (4)	3 (3)	3 (3)	4 (2)	4 (2)
		0.2~0.4	2 (4)	3 (3)	4 (2)	4 (2)	4 (2)
		0~0.2	3 (3)	4 (2)	5 (1)	5 (1)	5 (1)
Resistance value of ecological land expansion			1	2	3	4	5
Resistance value of built-up land expansion			5	4	3	2	1
Topography			Mountain	-	Hills	-	Plain
Soil erosion sensitivity	Slope (degree)		>25	15~25	8~15	3~8	0~3
	Vegetation coverage (%)		80~100	60~80	40~60	20~40	0~20
	Soil type		Skeleton soil	Alluvial soil, brown soil, purple soil	Yellow cinnamon soil, lime soil	-	Paddy soil
	Precipitation (mm)		1000~1200	900~1000	<900	-	-
Ecological function			Nature reserve, forest park	Basic farmland	-	-	Others
Landscape type			Forest, water	Farmland, grassland	Shrub	Bare land	Built- up land

#### Assessment of land suitability

The MCR model was used to find the minimum cumulative resistance surface of two expansion type. This study assumes the change of land type as the result of mutual competition between ecological land expansion and built-up land expansion. To reflect the spatial demand competitive relationship between ecological land and built-up land, the difference between the minimum cumulative resistance of ecological land expansion and the minimum cumulative resistance of built-up land expansion was calculated in a GIS environment. The formula is as follows:

$${MCR}_{diff}={MCR}_{ecolo}-{MCR}_{built}$$

where *MCR*_ecolo_ and the *MCR*_built_ indicate the minimum cumulative resistance of ecological land expansion and built-up land expansion, and the *MCR*_diff_ indicates the difference between the two minimum cumulative resistances.

**Fig 7:**
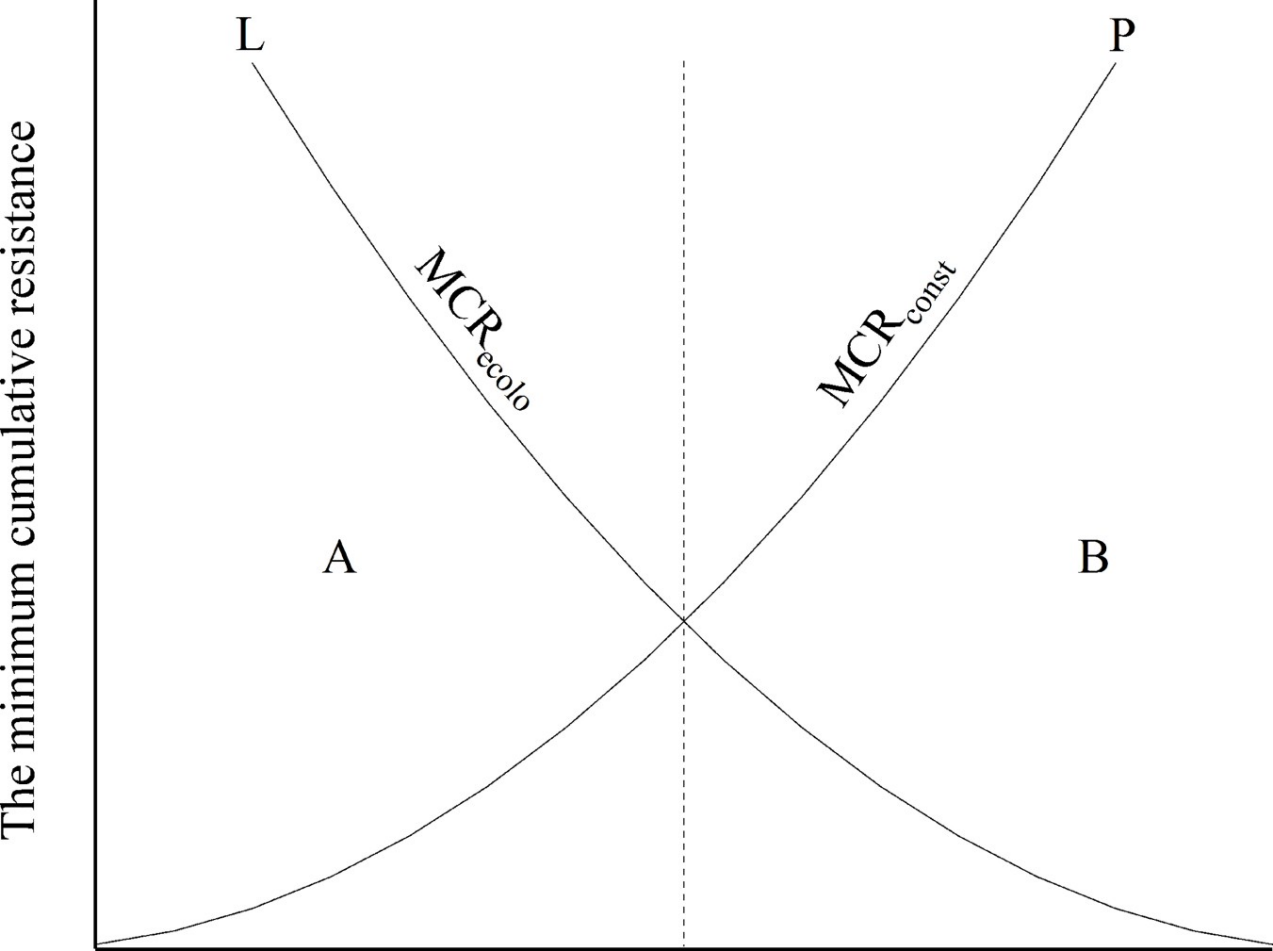
Balance of minimum cumulative resistance between ecological land and built-up land. [57].

In Fig 7, Curve L and Curve P indicate the minimum cumulative resistance of ecological land expansion and built-up land expansion, respectively. Positive *MCR*_diff_ in the A area means that the expansion resistance of ecological land is greater than the expansion resistance of built-up land while negative *MCR*_diff_ in the B area means the opposite. In other words, a smaller *MCR*_diff_ suggests a more suitable area is for the expansion of ecological land and a larger the *MCR*_diff_ suggests a more suitable area for the expansion of built-up land. Arranged in ascending order of *MCR*_diff_, the natural breaks (Jenks) classification method was used to divide the negative *MCR*_diff_ into ten levels, i.e., 1 to 10 levels, representing a gradual decrease in the suitability of ecological land expansion. Similarly, the positive *MCR*_diff_ was divided into 10 levels, i.e., 11 to 20 levels, representing to a gradual increase in the suitability of built-up land expansion. The minimum cumulative resistance surface of ecological land and built-up land and minimum cumulative resistance difference surface can be found in S1 Fig.

### Model calibration and prediction

As the most important step in the SLEUTH modeling process [25], model calibration aims to derive an optimal combination of the five coefficients. Using the earliest urban extent layer as the seed layer, the optimal coefficient combination is used to effectively simulate the urban growth during a historical period. Model calibration is implemented through a multi-stage, automated, and sequential process using a forced Monte Carlo iterative method [60]. The calibration process is divided into three phases: coarse calibration, fine calibration, and final calibration. First, users determine the iteration interval and iteration step size of the coefficients in the coarse calibration phase. Then each possible coefficient combination is tested for its ability to reproduce the historical growth patterns that took place between the input data years [61]. In the fine calibration and final calibration phase, based on the coarse calibration results, the range of the model coefficients and the iteration step size are sequentially reduced, and the optimal coefficients values are finally identified.

In order to assess the degree of fitting between simulated urban growth and actual urban growth, the model produces eight least-squares regression estimates for the calibration of each coefficient combination, i.e. Compare, Population, Edges, Clusters, Lee Sallee, Slope, X-mean, and Y-mean (descriptions given in Table 3) [26]. For each set of parameter combinations in the Monte Carlo iteration, the model counts the measurements of the simulated urban extent of the control year in its time series. The measurements of the simulated urban extent are compared to the actual urban extent to produce multiple least squares regression measurements [29]. Lee Sallee, the ratio of the intersection and the union of the simulated and actual urban extent, is a measurement of spatial fit [62]. These statistics are calculated internally in the model and output as log files. Despite a variety of methods for reducing the iteration interval, none has been ever considered the most effective [16]. In this study, the optimal coefficient combination of each calibration phase was selected by the Optimal SLEUTH Metric (OSM) parameter. The OSM parameter (product of seven parameters of Compare, Population, Edges, Clusters, Slope, X-mean, and Y-mean) developed by Dietzel and Clarke can provide reliable calibration results for the SLEUTH model [6,12]. The calibration process of this study are described as follows:

- Coarse calibration. The iteration range of coefficients was set from 0 to 100, the step size was set at 25, and the number of the Monte Carlo iterations was set at 4. The results of the calibration were sorted in ascending order of the OSM parameter, and the top three highest scoring results were used to narrow the iteration interval.
- Fine calibration. The iteration interval was set based on the coarse calibration result. The step size was set at 5~15, and the number of the Monte Carlo iterations was set at 8. The coarse calibration work was repeated to get a new narrower iteration interval.
- Final calibration. The final iteration interval was obtained from the fine calibration results. The step size was set at 1~2, and the number of the Monte Carlo iterations was set at 12. The coefficient combination with the highest OSM parameter was selected as the optimal coefficient combination.

**Table 3:** The parameters assessing the model calibration for the three scenarios. [63].

Parameter	Description	Scenario 1	Scenario 2	Scenario 3
Compare	Modeled population for final year/actual population for final year	0.999	0.723	0.952
Population	Least squares regression score for modeled urbanization compared to actual urbanization for the control years	0.997	0.999	0.997
Edges	Least squares regression score for modeled urban edge count compared to actual urban edge count for the control years	0.836	0.854	0.831
Cluster	Least squares regression score for modeled urban clustering compared to known urban clustering for the control years∉	0.996	0.999	0.992
Lee-Salle	A shape index, a measure of spatial fit between the model’s growth and the known urban extent for the control years	0.349	0.431	0.380
Slope	Least squares regression of average slope for modeled urbanized cells compared to the average slope of known urban cells for the control years	0.957	0.933	0.947
X-mean	Least squares regression of average x-values for modeled urbanized cells compared to average x-values of known urban cells for the control years	0.993	0.998	0.998
Y-mean	Least squares regression of average y-values for modeled urbanized cells compared to average y-values of known urban cells for the control years	0.999	0.986	0.976

Due to the self-modifying nature of the SLEUTH model, the initial coefficient combination of the model is changed during the simulation process. Therefore, the optimal coefficient combination for obtaining the final calibration was used as the starting and stopping values for the iteration interval, and 100 Monte Carlo iterations were performed to obtain the optimal results [15,29].

The calibration process resulted in the optimal coefficient combination for urban growth. These optimal coefficient combinations were incorporated into the prediction module of the model as initialization coefficients. In the model prediction phase, the 2015 urban built-up land was used as the seed layer, the number of the Monte Carlo iterations was set at 100, and the urbanization probability threshold was set at 50%~100%. Finally, the prediction module of the SLEUTH model was used to predict urban growth from 2015 to 2040 in the three growth scenarios.

## Results

### Model calibration

Using the historical data from 2000 to 2015, model calibration was separately performed for the three scenarios, each of which had its own exclusion layer. The results of model calibration for the three scenarios are given in Table 4, Table 5, and Table 6. The optimal coefficient combination was 100, 75, 64, 39, and 73 for dispersion, breed, spread, slope, and road gravity in Scenario 1, 98, 99, 100, 2, and 82 in Scenario 2, and was 99, 100, 100, 1, and 62 in Scenario 3. Parameter values for the three phases of the calibration mode (coarse, fine, final) for each scenario are presented in Tables 3–5, which lists the highest OSM value from thousands of model calibration runs. The OSM parameter increased from 0.785 to 0.788 in Scenairo1, from 0.564 to 0.576 in Scenario 2, and from 0.710 to 0.716 in Scenario 3. Each round of calibration produced a higher SOM value, indicating that the model calibration was valid.

**Table 4:** Model calibration for the historical growth scenarios.

Historical growth scenarios							
Model coefficient	Coarse		Fine		Final		Optimal coefficient combination
	Number of iterations = 4		Number of iterations = 8		Number of iterations = 12		
	OSM parameter = 0.785		OSM parameter = 0.787		OSM parameter = 0.788		
	Range	Step	Range	Step	Range	Step	
Dispersion	0~100	25	75~100	5	95~100	1	100
Breed	0~100	25	75~100	5	75~80	1	75
Spread	0~100	25	50~100	10	50~60	2	64
Slope	0~100	25	25~50	5	45~50	1	39
Road gravity	0~100	25	50~100	10	60~90	6	73

**Table 5:** Model calibration for the urban planning growth scenario.

Urban planning growth scenario							
Model coefficient	Coarse		Fine		Final		Optimal coefficient combination
	Number of iterations = 4		Number of iterations = 8		Number of iterations = 12		
	OSM parameter = 0.564		OSM parameter = 0.564		OSM parameter = 0.567		
	Range	Step	Range	Step	Range	Step	
Dispersion	0~100	25	75~100	5	95~100	1	98
Breed	0~100	25	75~100	5	95~100	1	99
Spread	0~100	25	75~100	5	95~100	1	100
Slope	0~100	25	25~50	5	1~10	2	2
Road gravity	0~100	25	50~100	10	70~85	3	82

**Table 6:** Model calibration for the land suitability growth scenario.

Land suitability growth scenario							
Model coefficient	Coarse		Fine		Final		Optimal coefficient combination
	Number of iterations = 4		Number of iterations = 8		Number of iterations = 12		
	OSM parameter = 0.710		OSM parameter = 0.714		OSM parameter = 0.719		
	Range	Step	Range	Step	Range	Step	
Dispersion	0~100	25	75~100	5	95~100	1	99
Breed	0~100	25	75~100	5	95~100	1	100
Spread	0~100	25	75~100	5	95~100	1	100
Slope	0~100	25	0~25	5	1~10	2	1
Road gravity	0~100	25	25~100	15	20~30	2	62

A practicable way to assess the accuracy of the SLETUH model, as done in most of previous studies, is examining whether the model can reproduce a similar urban extent of a historical year. Table 3 shows the assessment of the model calibration for the three scenarios. The Comparison and Population parameters represent the similarity between the actual urban area and the predicted urban area based on the final year. The values of the Compare parameter for Scenario 1 and Scenario 3 reached 0.999 and 0.952 respectively, both higher than that for Scenario 2 (0.72). The values of the Population parameter were all above 0.99. The values of the Edges parameter, indicating the correlation between the urban edge of the actual year and the urban edge of the simulated year, were 0.83,0.85,0.83, and the values of the Clusters parameter were all above 0.99 for the three scenarios, showing the capability of the model to simulate accurately both the changes of urban shape and urban clusters. The values of the Lee-Salle parameter were overall slightly low (ranging from 0.35 to 0.43) but acceptable (0.3 ~ 0.7). The values of the X-mean parameter and Y-mean parameter were all above 0.97, showing the high correlation between the urbanized cells location of the actual year and the urban of the simulated year. The results of each parameters evaluation show that the calibration results have satisfactory simulation effects in terms of the urban area, urban shape, urban cluster, urban location, etc., which allows us to be confident in using the SLEUTH model to predict the urban extent in the year of 2040.

### Model prediction

**Fig 8:**
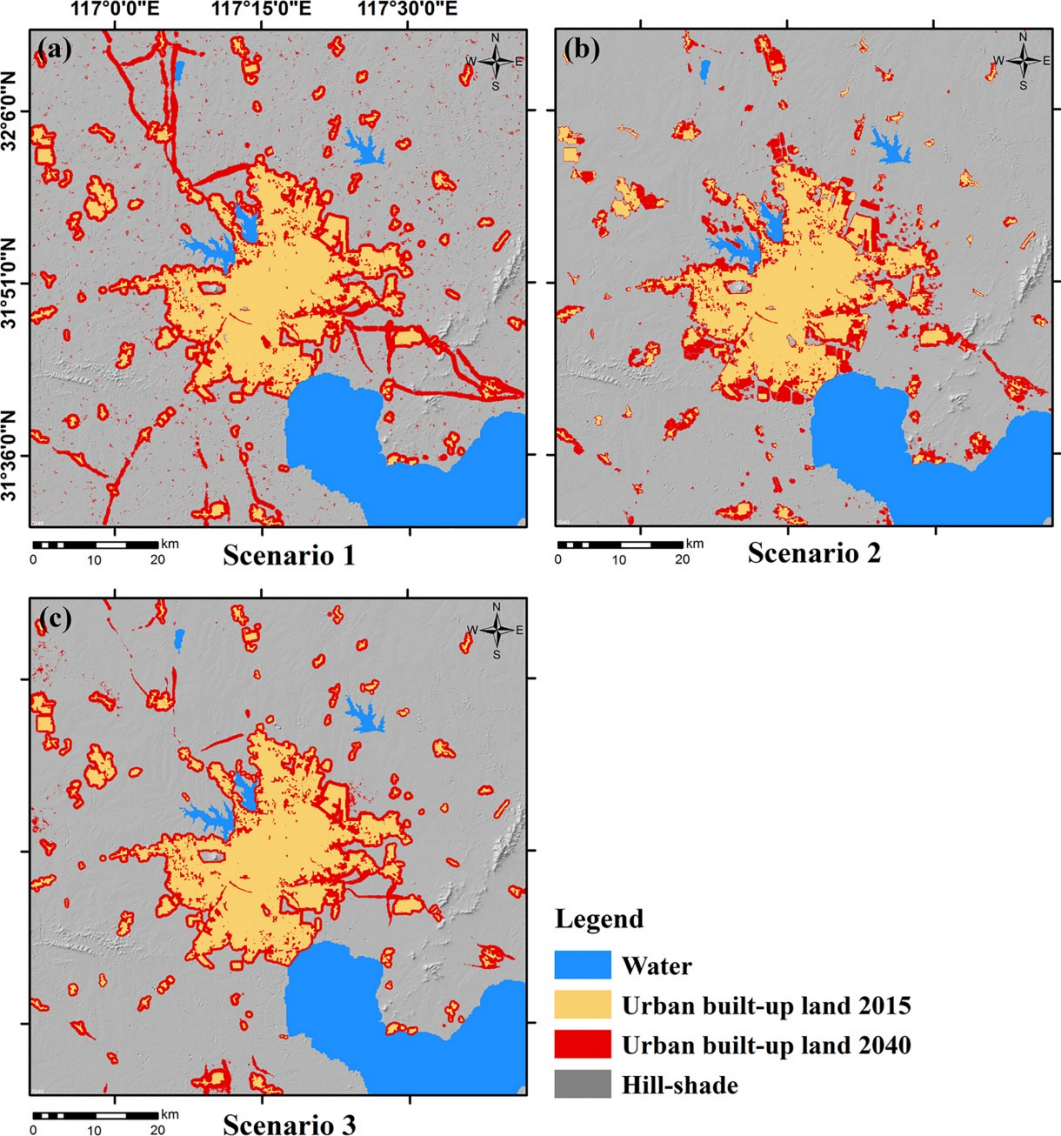
Urban built-up area predicted for three scenarios in 2040. (a) Scenario 1, the historical growth scenario; (b) Scenario 2, the urban planning growth scenario; (c) Scenario 3, the land suitability growth scenario.

Using the 2015 urban area as the seed layer, we used the SLEUTH model to predict Hefei’s urban growth in 2040 in three different urban growth scenarios (Fig 8).

In the historical growth scenario (Fig 8A), the urban built-up land area in 2040 would increase to 1434 km^2^ and gain 748 km^2^ newly added built-up land compared with 2015, with an average annual urban growth rate of 3.12%. Such an increase would be at the expense of other types of land, e.g., farmland decreased by 13.89%, forest land decreased by 4.32%, and grassland decreased by 15.67% (Table 7). In this scenario, the exclusion layer only set water as non-urbanizable areas, with few restrictions on urban growth. The urban growth would be based entirely on the trends of historical growth. The urban boundary expansion would be obvious, and the expansion distance would be uniform. Newly added urban built-up land of large areas would occur along major traffic roads while much newly added urban built-up land of small areas would be scattered non-water areas.

**Table 7:** Urban built-up land area predicted for 2040 in the three scenarios and the source of the newly added urban built-up land.

Scenarios	Urban built-up land (km^2^)	Average annual urban growth rate (%)	Obtained by occupying farmland (km^2^)	Obtained by occupying forestland (km^2^)	Obtained by occupying grassland (km^2^)
Scenario 1	1434	3.12	567.457 (13.89%)	4.572 (4.32%)	10.192 (15.67%)
Scenario 2	1190	2.32	355.327 (8.70%)	3.146 (2.98%)	6.901 (10.61%)
Scenario 3	1217	2.41	388.141 (9.49%)	2.415 (2.28%)	7.581 (11.65%)

Note: The percentages indicate the contributions of each source to newly added built-up land. The average annual urban growth rate $R=\sqrt[{24}]{S_{2040}/S_{2015}}-1$, where *S*_2015_ and *S*_2040_ represent the urban built-up land area of 2015 and 2040, respectively.

In the urban planning growth scenario (Fig 8B), by 2040, the urban built-up area would increase to 1190 km^2^, an increase of 504 km^2^ over 2015, with an average annual urban growth rate of 2.32%. Compared with the prediction from Scenario 1, it is clear that less farmland, forest, and grassland would be lost. According to Hefei’s 2006–2020 general land use planning map, 46.05% of the newly added urban built-up land would be from the LPC and LCPC. Newly added urban built-up land would be concentrated in the hot spots for urban development Feidong Economic Development Area (east of the main urban area), the Shushan Economic Development Area (west of the main urban area), and the Hefei Economic and Technological Development Area (south of the main urban area).

In the land suitability growth scenario (Fig 8C), the urban built-up land area in 2040 would be 1217 km^2^—there would be 531 km^2^ of newly added urban built-up land with an average annual urban growth rate of 2.41%. The contributions of farmland, forestland, and grassland to the newly added urban built-up land in Scenario 3 are quite similar to those in Scenario 2. Due to the limitation of ecological suitability, the marginal expansion of urban would be not uniform. As far as the main urban area is concerned, the eastern and northeastern borders would be more vigorously expanded. The impact of the road network on urban growth would be significant but, unlike Scenario 1, the growth along the road network in this scenario would occur mostly in the suburbs of Hefei.

## Discussion

### Optimal coefficient combination

The optimal coefficient combination reveals the historical expansion patterns and constraints of the urban area and determines how the future urban growth would expand [13]. In previous research, the optimal coefficient combination from a simple exclusion layer, like Scenario 1, is often used by other complex scenarios [15,46]. In this case, the simulation from complex scenarios may be underestimated [64]. Thus, the three exclusion layers in this study were separately calibrated and predicted. The calibration results show that the optimal coefficient combination varies with different the scenario. In other words, model calibration is sensitive to the exclusion layer.

Between 2000 and 2015, Hefei accelerated the revival of existing townships and construction of new ones. This explains why the values of the Dispersion coefficients were high for all scenarios (Tables 3–5). According to the urban development strategy of Hefei, four urban groups were gradually formed in the east, north, west, and southwest of the main urban area of Hefei. This urban spatial structure was evident in the urban extent layer of the year 2010 (Fig 3C). The high values of the Breed coefficient reflect the new growth center growth as the main way of urban growth. Scenario 1 did not limit the expansion of the urban edge. The urban margins of Scenario 2 and Scenario 3, however, were set to different urbanization exclusion probabilities. Edge growth was greatly limited. Thus, the SLEUTH model countered the limitations of the exclusion layer on historical expansion trends in Scenario 2 and Scenario 3 by significantly increasing the Spread coefficient. Similarly, the Slope coefficient in Scenario 2 and Scenario 3 had dropped to a very low level to increase the probability of urbanization. The Slope coefficient of the three scenarios was relatively low, which shows that urban extent tends to grow in flat areas, and terrain is not a major obstacle to urban growth. The high values of the Road Gravity coefficient in the three scenarios implied the strong attraction of transportation to urban growth.

### Impact of exclusion layer on prediction

As shown in Table 7 and Fig 8, the exclusion layer has a significant impact on the rate and spatial structure of urban growth. The speed of urban simulation growth under the three scenarios is lower than the historical expansion speed. In particular, the expansion of urban built-up land in Scenario 2 and Scenario 3 would be regulated, and farmland, forestland, and grassland would be overall protected. In terms of urban spatial pattern, the urban area predicted from Scenario 1 would be clearly not ideal, because the impact of spontaneous growth and road network on urban growth would be overestimated. Unconstrained exclusion layers in some cases may lead to unreasonable predictions from the SLEUTH model.

Nevertheless, the exclusion layers of Scenario 2 and Scenario 3 would direct urban growth from the perspective of urban planning and land suitability. Scenario 2 would clearly illustrate the hot spots of future urban development, such as the southwest, north, and east parts of the main urban area. To some extent, we think that Scenario 2 agrees with planning policies of urban growth. Although the optimal coefficient combination of Scenario 2 and Scenario 3 were not remarkably different, the prediction was contrasting due to the different exclusion layers. Urban growth in Scenario 2 would be limited to areas of high suitability for built-up land. In the MCR-based land suitability assessment, the spatial distance is regarded as an important factor for the expansion of the source. The closer to the built-up land source, the higher the suitability of the built-up land expansion. Therefore, the edge growth of Scenario 3 would be obvious, and the spatial structure of urban growth would also be closer.

In previous researches, the setting of the exclusion layer was relatively simplified as they usually assigned a few different exclusion probabilities. Examples include the simulation of Busan in South Korea where the exclusion probabilities for the three levels were 100% for a greenbelt and 75%, and 0% for two levels of legal conservation areas, respectively [48], and a study of Shanghai in China where the exclusion probability was divided into three levels, 100% for water areas and wetlands, 80% for farmlands, and 60% for grassland [65]. By dividing the MCR-based land suitability assessment map into 20 grades in our study, the exclusion probability was however continuously changed, from 0% to 100%, and the exclusion layer that we generated for Scenario 3 would better characterize the suitability of land for urbanization. The land suitability urban growth would avoid the scattered distribution of small-area built-up land as well as excessive protection of farmland ecological land.

### Limitations

Using a decreased data resolution (60 m) facilitate data processing, but it might lower the accuracy of urban growth prediction. Although urban planning was incorporated into the exclusion layer for Scenario 2 to represent the role of governmental policies in urban growth, it did not consider may other socioeconomic factors, e.g., population, agricultural land value and industrialization [66,67]. As such, adding more urban growth drivers to the SLEUTH model can be a direction for future work.

## Conclusions

Using the SLEUTH model, we predicted the expansion of urban built-up land in Hefei from 2015 to 2040 in three different urban growth scenarios, each of which had its own exclusion layer and optimal coefficient combination. Results of each scenario were compared and analyzed. The main findings and conclusions are summarized as follows:

- Compared with the other two scenarios, the historical growth scenario produces an improper urban built-up land distribution, the highest urban growth rate, and the highest loss of farmland, forestland, and grassland. It suggests that exclusion layers without effective restrictions may result in unreasonable urban growth predictions.
- Despite a medium urban growth rate, the urban planning growth scenario would occupy the least amount of other land. Based on the general land use map, the urban growth prediction took the governmental policies into account and could reveal the development hotspots in urban planning.
- The land suitability scenario prediction was the result of the trade-off between ecological land and built-up land as it used the MCR-based land suitability assessment result. This scenario predicts the lowest urban growth rate and a medium loss of farmland, forestland, and grassland. It would help to form a compact urban space and avoid excessive protection of farmland and ecological land.

In summary, the urban built-up land in Hefei remains in a rapid growth stage in the next two decades. The expansion of built-up land would be at the expense of main farmland, and to a lesser extent, of ecological land. Coordinating the contradiction between urban growth and the protection of farmland and ecological land would be the key to sustainable urban development in Hefei. At the same time, the planning of urban space should be given importance. Findings of this study provide useful insights into the characteristics of urban development and formulating urban development plans.

## Supporting information

S1 Fig Results of the minimum cumulative resistance evaluation. (a) Minimum cumulative resistance surface of ecological land; (b) Minimum cumulative resistance surface of built-up land; (c) Minimum cumulative resistance difference surface.
(TIF)

## References

[1] Zhou X, Chen H. Impact of urbanization-related land use land cover changes and urban morphology changes on the urban heat island phenomenon. Sci Total Environ. 2018;635: 1467–1476. 10.1016/j.scitotenv.2018.04.091 29710597

[2] Estoque RC, Murayama Y. Landscape pattern and ecosystem service value changes: Implications for environmental sustainability planning for the rapidly urbanizing summer capital of the Philippines. Landsc Urban Plan. 2013;116: 60–72. 10.1016/j.landurbplan.2013.04.008

[3] Bai Y, Deng X, Jiang S, Zhang Q, Wang Z. Exploring the relationship between urbanization and urban eco-efficiency: Evidence from prefecture-level cities in China. J Clean Prod. 2018;195: 1487–1496. 10.1016/j.jclepro.2017.11.115

[4] Hu Z, Lo CP. Modeling urban growth in Atlanta using logistic regression. Comput Environ Urban Syst. 2007;31(6): 667–688. 10.1016/j.compenvurbsys.2006.11.001

[5] Vermeiren K, Van Rompaey A, Loopmans M, Serwajja E, Mukwaya P. Urban growth of Kampala, Uganda: Pattern analysis and scenario development. Landsc Urban Plan. 2012;106(2): 199–206. 10.1016/j.landurbplan.2012.03.006

[6] Alsharif AAA, Pradhan B. Urban sprawl analysis of Tripoli Metropolitan City (Libya) using remote sensing data and multivariate logistic regression model. J Indian Soc Remote Sens. 2014;42(1): 149–163. 10.1007/s12524-013-0299-7

[7] Hosseinali F, Alesheikh AA, Nourian F. Agent-based modeling of urban land-use development, case study: Simulating future scenarios of Qazvin city. Cities. 2013;31: 105–113. 10.1016/j.cities.2012.09.002

[8] Santé I, García AM, Miranda D, Crecente R. Cellular automata models for the simulation of real-world urban processes: A review and analysis. Landsc Urban Plan. 2010;96(2): 108–122. 10.1016/j.landurbplan.2010.03.001

[9] Triantakonstantis D, Mountrakis G. Urban growth prediction: A review of computational models and human perceptions. J Geogr Inf Syst. 2012;4(6): 555–587. 10.4236/jgis.2012.46060

[10] Musa SI, Hashim M, Reba MNM. A review of geospatial-based urban growth models and modelling initiatives. Geocarto Int. 2017;32(8): 813–833. 10.1080/10106049.2016.1213891

[11] Aburas MM, Ho YM, Ramli MF, Ash’aari ZH. The simulation and prediction of spatio-temporal urban growth trends using cellular automata models: A review. Int J Appl Earth Obs Geoinf. 2016;52: 380–389. 10.1016/j.jag.2016.07.007

[12] Liu Y, Hu Y, Long S, Liu L, Liu X. Analysis of the effectiveness of urban land-use-change models based on the measurement of spatio-temporal, dynamic urban growth: A cellular automata case study. Sustain. 2017;9(5): 1–15. 10.3390/su9050796

[13] Hua L, Tang L, Cui S, Yin K. Simulating urban growth using the SLEUTH model in a coastal peri-urban district in China. Sustain. 2014;6(6): 3899–3914. 10.3390/su6063899

[14] Zheng Q, Yang X, Wang K, Huang L, Shahtahmassebi AR, Gan M, et al Delimiting urban growth boundary through combining land suitability evaluation and cellular automata. Sustain. 2017;9(12): 2213 10.3390/su9122213

[15] Rafiee R, Mahiny AS, Khorasani N, Darvishsefat AA, Danekar A. Simulating urban growth in Mashad City, Iran through the SLEUTH model (UGM). Cities. 2009;26(1): 19–26. 10.1016/j.cities.2008.11.005

[16] Dietzel C, Clarke KC. Replication of spatio-temporal land use patterns at three levels of aggregation by an urban cellular automata. 6th Int Conf Cell Autom Res Ind ACRI 2004 2004; 523–532. 10.1007/978-3-540-30479-1_54

[17] Chen Y, Liu X, Li X. Calibrating a Land Parcel Cellular Automaton (LP-CA) for urban growth simulation based on ensemble learning. Int J Geogr Inf Sci. 2017;31(12): 2480–2504. 10.1080/13658816.2017.1367004

[18] Al-Darwish Y, Ayad H, Taha D, Saadallah D. Predicting the future urban growth and it’s impacts on the surrounding environment using urban simulation models: Case study of Ibb city–Yemen. Alexandria Eng J. 2018;57(4): 2887–2895. 10.1016/j.aej.2017.10.009

[19] Waddell P. UrbanSim: Modeling urban development for land use, transportation, and environmental planning. J Am Plan Assoc. 2002;68(3): 297–314. 10.1080/01944360208976274

[20] Deng Z, Zhang X, Li D, Pan G. Simulation of land use/land cover change and its effects on the hydrological characteristics of the upper reaches of the Hanjiang Basin. Environ Earth Sci. 2015;73(3): 1119–1132. 10.1007/s12665-014-3465-5

[21] Yang Q, Li X, Shi X. Cellular automata for simulating land use changes based on support vector machines. Comput Geosci. 2008;34(6): 592–602. 10.1016/j.cageo.2007.08.003

[22] Kamusoko C, Gamba J. Simulating urban growth using a Random Forest-Cellular Automata (RF-CA) model. ISPRS Int J Geo-Information. 2015;4(2): 447–470. 10.3390/ijgi4020447

[23] Liang X, Liu X, Li D, Zhao H, Chen G. Urban growth simulation by incorporating planning policies into a CA-based future land-use simulation model. Int J Geogr Inf Sci. 2018;32(11): 2294–2316. 10.1080/13658816.2018.1502441

[24] Clarke KC, Hoppen S, Gaydos L. A self-modifying cellular automaton model of historical urbanization in the San Francisco Bay area. Environ Plan B Plan Des. 1997;24(2): 247–261. 10.1068/b240247

[25] Silva EA, Clarke KC. Calibration of the SLEUTH urban growth model for Lisbon and Porto, Portugal. Comput Environ Urban Syst. 2002;26(6): 525–552. 10.1016/S0198-9715(01)00014-X

[26] Dietzel C, Clarke KC. Toward optimal calibration of the SLEUTH land use change model. Trans GIS. 2007;11(1): 29–45. 10.1111/j.1467-9671.2007.01031.x

[27] Jantz CA, Goetz SJ, Donato D, Claggett P. Designing and implementing a regional urban modeling system using the SLEUTH cellular urban model. Comput Environ Urban Syst. 2010;34(1): 1–16. 10.1016/j.compenvurbsys.2009.08.003

[28] Li F, Wang L, Chen Z, Clarke KC, Li M, Jiang P. Extending the SLEUTH model to integrate habitat quality into urban growth simulation. J Environ Manage. 2018;217: 486–498. 10.1016/j.jenvman.2018.03.109 29631238

[29] Serasinghe Pathiranage IS, Kantakumar LN, Sundaramoorthy S. Remote sensing data and SLEUTH urban growth model: As decision support tools for urban planning. Chinese Geogr Sci. 2018;28(2): 274–286. 10.1007/s11769-018-0946-6

[30] Bihamta N, Soffianian A, Fakheran S, Gholamalifard M. Using the SLEUTH urban growth model to simulate future urban expansion of the Isfahan metropolitan area, Iran. J Indian Soc Remote Sens. 2015;43(2): 407–414. 10.1007/s12524-014-0402-8

[31] Sakieh Y, Salmanmahiny A, Jafarnezhad J, Mehri A, Kamyab H, Galdavi S. Evaluating the strategy of decentralized urban land-use planning in a developing region. Land use policy. 2015;48: 534–551. 10.1016/j.landusepol.2015.07.004

[32] Han H, Hwang YS, Ha SR, Kim BS. Modeling future land use scenarios in South Korea: Applying the IPCC special report on emissions scenarios and the SLEUTH model on a local scale. Environ Manage. 2015;55(5): 1064–1079. 10.1007/s00267-015-0446-8 25588808

[33] Butsch C, Kumar S, Wagner P, Kroll M, Kantakumar L, Bharucha E, et al Growing ‘smart’? Urbanization processes in the Pune Urban Agglomeration. Sustain. 2017;9(12): 2335 10.3390/su9122335

[34] Yan Y, Zhou R, Ye X, Zhang H, Wang X. Suitability evaluation of urban construction land based on an approach of vertical-horizontal processes. ISPRS Int J Geo-Information. 2018;7(5): 198 10.3390/ijgi7050198

[35] Chen M, Gong Y, Lu D, Ye C. Build a people-oriented urbanization: China’s new-type urbanization dream and Anhui model. Land use policy. 2019;80: 1–9. 10.1016/j.landusepol.2018.09.031

[36] Chen M, Liu W, Lu D. Challenges and the way forward in China’s new-type urbanization. Land use policy. 2016;55: 334–339. 10.1016/j.landusepol.2015.07.025

[37] Huang C, Davis LS, Townshend JRG. An assessment of support vector machines for land cover classification. Int J Remote Sens. 2002;23(4): 725–749. 10.1080/01431160110040323

[38] Poursanidis D, Chrysoulakis N, Mitraka Z. Landsat 8 vs. Landsat 5: A comparison based on urban and peri-urban land cover mapping. Int J Appl Earth Obs Geoinf. 2015;35: 259–269. 10.1016/j.jag.2014.09.010

[39] Zhou X, Li L, Chen L, Liu Y, Cui Y, Zhang Y, et al Discriminating urban forest types from Sentinel-2A image data through linear spectral mixture analysis: A case study of Xuzhou, East China. Forests. 2019;10(6): 478 10.3390/f10060478

[40] Li L, Bakelants L, Solana C, Canters F, Kervyn M. Dating lava flows of tropical volcanoes by means of spatial modeling of vegetation recovery. Earth Surf Process Landforms. 2018;43(4): 840–856. 10.1002/esp.4284

[41] van der Linden S, Rabe A, Held M, Jakimow B, Leitão P, Okujeni A, et al The EnMAP-Box—A toolbox and application programming interface for EnMAP data processing. Remote Sens. 2015;7(9): 11249–11266. 10.3390/rs70911249

[42] Cui Y, Li L, Chen L, Zhang Y, Cheng L, Zhou X, et al Land-use carbon emissions estimation for the Yangtze River Delta Urban Agglomeration using 1994–2016 Landsat image data. Remote Sens. 2018;10(9): 1334 10.3390/rs10091334

[43] Li L, Solana C, Canters F, Kervyn M. Testing random forest classification for identifying lava flows and mapping age groups on a single Landsat 8 image. J Volcanol Geotherm Res. 2017;345: 109–124. 10.1016/j.jvolgeores.2017.07.014

[44] Kuo HF, Tsou KW. Modeling and simulation of the future impacts of urban land use change on the natural environment by SLEUTH and cluster analysis. Sustain. 2017;10(2): 72 10.3390/su10010072

[45] Yang X, Lo CP. Modelling urban growth and landscape changes in the Atlanta Metropolitan Area. Int J Geogr Inf Sci. 2003;17(5): 463–488. 10.1080/1365881031000086965

[46] Nigussie TA, Altunkaynak A. Modeling urbanization of Istanbul under different scenarios using SLEUTH urban growth model. J Urban Plan Dev. 2017;143(2): 04016037 10.1061/(ASCE)UP.1943-5444.0000369

[47] Wu X, Hu Y, He HS, Bu R, Onsted J, Xi F. Performance evaluation of the SLEUTH model in the Shenyang Metropolitan Area of Northeastern China. Environ Model Assess. 2009;14(2): 221–230. 10.1007/s10666-008-9154-6

[48] Kim J, Park S. Simulating the impacts of the greenbelt policy reform on sustainable urban growth: The case of Busan Metropolitan Area. J Korean Soc Surv Geod Photogramm Cartogr. 2015;33(3): 193–202. 10.7848/ksgpc.2015.33.3.193

[49] Ministry of Land and Resources of the PRC. Guideline for the county-level general land use planning (TD/T 1024–2010) Beijing: Standards Press of China; 2010. (in Chinese)

[50] Tong H, Shi P, Bao S, Zhang X, Nie X. Optimization of urban land development spatial allocation based on ecology-economy comparative advantage perspective. J Urban Plan Dev. 2018;144(2): 05018006 10.1061/(ASCE)UP.1943-5444.0000444

[51] Ying C, Ling H, Kai H. Change and optimization of landscape patterns in a basin based on remote sensing images: A case study in China. Polish J Environ Stud. 2017;26(5): 2343–2353. 10.15244/pjoes/70007

[52] Knaapen JP, Scheffer M, Harms B. Estimating habitat isolation in landscape planning. Landsc Urban Plan. 1992;23(1): 1–16. 10.1016/0169-2046(92)90060-D

[53] Liu X, Shu J, Zhang L. Research on applying minimal cumulative resistance model in urban land ecological suitability assessment: as an example of Xiamen City. Acta Ecol Sin. 2010 2010;30(2): 421–428. (in Chinese)

[54] Yu K. Security patterns and surface model in landscape ecological planning. Landsc Urban Plan. 1996;36(1): 1–17. 10.1016/S0169-2046(96)00331-3

[55] Gao Y, Ma L, Liu J, Zhuang Z, Huang Q, Li M. Constructing ecological networks based on habitat quality assessment: A case study of Changzhou, China. Sci Rep. Nature Publishing Group; 2017;7: 1–11. 10.1038/srep46073 28393879 PMC5385553

[56] Liu G, Liang Y, Cheng Y, Wang H, Yi L. Security patterns and resistance surface model in urban development: Case study of Sanshui, China. J Urban Plan Dev. 2017;143(4): 05017011 10.1061/(ASCE)UP.1943-5444.0000402

[57] Li F, Ye Y, Song B, Wang R. Evaluation of urban suitable ecological land based on the minimum cumulative resistance model: A case study from Changzhou, China. Ecol Modell. 2015;318: 194–203. 10.1016/j.ecolmodel.2014.09.002

[58] Yu Q, Yue D, Wang J, Zhang Q, Li Y, Yu Y, et al The optimization of urban ecological infrastructure network based on the changes of county landscape patterns: a typical case study of ecological fragile zone located at Deng Kou (Inner Mongolia). J Clean Prod. 2017;163: S54–S67. 10.1016/j.jclepro.2016.05.014

[59] Ye Y, Su Y, Zhang H ou, Liu K, Wu Q. Construction of an ecological resistance surface model and its application in urban expansion simulations. J Geogr Sci. 2015;25(2): 211–224. 10.1007/s11442-015-1163-1

[60] Sakieh Y, Salmanmahiny A. Treating a cancerous landscape: Implications from medical sciences for urban and landscape planning in a developing region. Habitat Int. 2016;55: 180–191. 10.1016/j.habitatint.2016.03.008

[61] Dietzel C, Clarke KC. Spatial differences in multi-resolution urban automata modeling. Trans GIS. 2004;8(4): 479–492. 10.1111/j.1467-9671.2004.00197.x

[62] Mitsova D, Shuster W, Wang X. A cellular automata model of land cover change to integrate urban growth with open space conservation. Landsc Urban Plan. 2011;99(2): 141–153. 10.1016/j.landurbplan.2010.10.001

[63] Onsted J, Clarke KC. The inclusion of differentially assessed lands in urban growth model calibration: A comparison of two approaches using SLEUTH. Int J Geogr Inf Sci. 2012;26(5): 881–898. 10.1080/13658816.2011.617305

[64] Akın A, Clarke KC, Berberoglu S. The impact of historical exclusion on the calibration of the SLEUTH urban growth model. Int J Appl Earth Obs Geoinf. 2014;27: 156–168. 10.1016/j.jag.2013.10.002

[65] Shi Y, Wu J, Shi S. Study of the simulated expansion boundary of the simulated expansion boundary of construction land in Shanghai based on a SLEUTH model. Sustain. 2017;9(6):876 10.3390/su9060876

[66] Li G, Sun S, Fang C. The varying driving forces of urban expansion in China: Insights from a spatial-temporal analysis. Landsc Urban Plan. 2018;174: 63–77. 10.1016/j.landurbplan.2018.03.004 33601670

[67] Deng X, Huang J, Rozelle S, Uchida E. Growth, population and industrialization, and urban land expansion of China. J Urban Econ. 2008;63(1): 96–115. 10.1016/j.jue.2006.12.006

