# Supplementary figures and images for: Urban growth simulation in different scenarios using the SLEUTH model: A case study of Hefei, East China
Source: PLoS One. 2019 Nov 7;14(11):e0224998. doi: 10.1371/journal.pone.0224998 (PMC6837527; doi:10.1371/journal.pone.0224998)

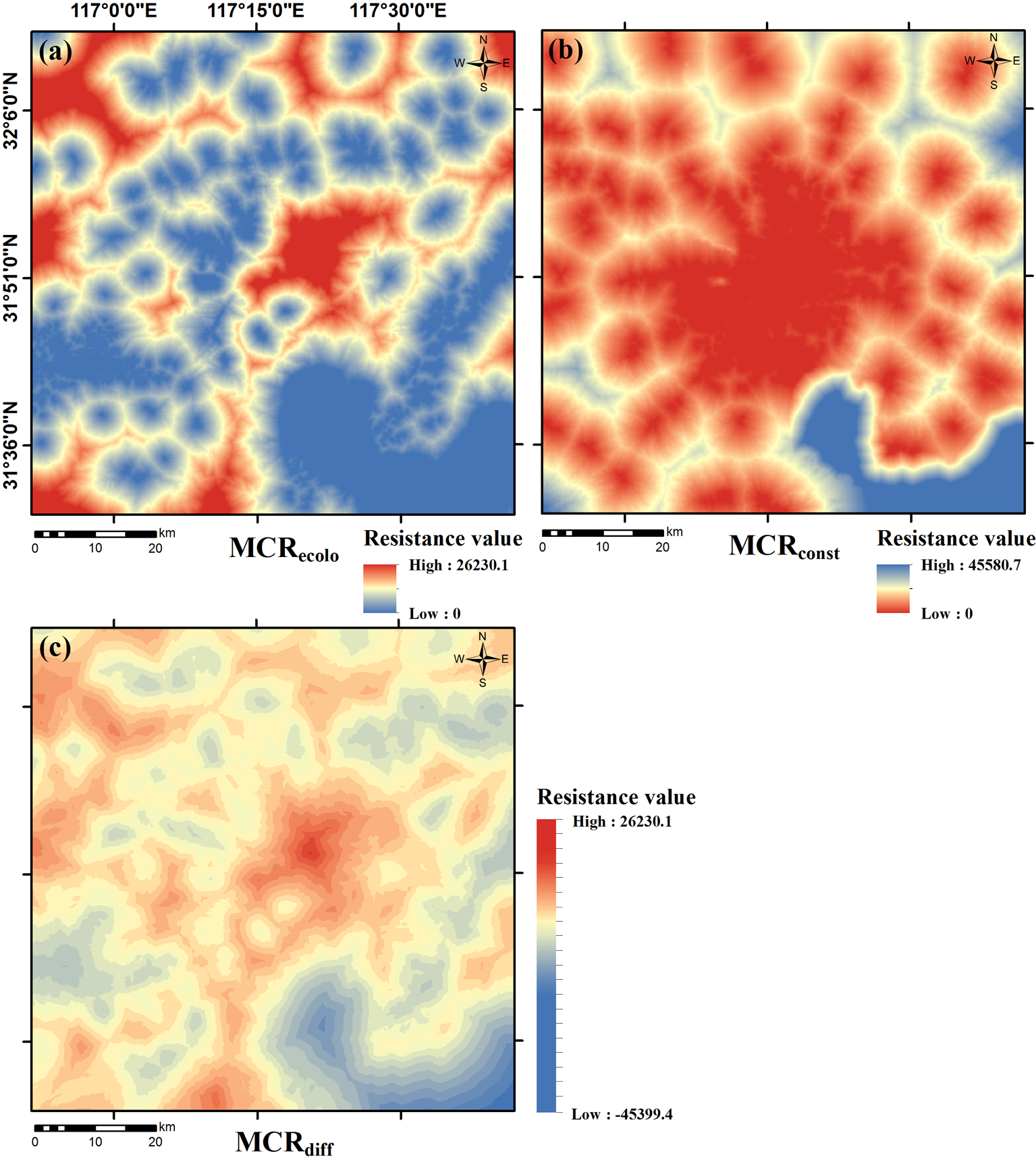

Supplement: S1 Fig — (a) Minimum cumulative resistance surface of ecological land; (b) Minimum cumulative resistance surface of built-up land; (c) Minimum cumulative resistance difference surface. (TIF) [file pone.0224998.s001.tif]
